# *Streptococcus agalactiae* in pregnant women: serotype and antimicrobial susceptibility patterns over five years in Eastern Sicily (Italy)

**DOI:** 10.1007/s10096-020-03992-8

**Published:** 2020-07-22

**Authors:** Carlo Genovese, Floriana D’Angeli, Valentina Di Salvatore, Gianna Tempera, Daria Nicolosi

**Affiliations:** 1 Department of Biomedical and Biotechnological Sciences, Section of Microbiology, University of Catania, Catania, 95123 Italy; 2 Nacture S.r.l, Spin-off University of Catania, Catania, 95123 Italy; 3 Department of Biomedical and Biotechnological Sciences, Section of Biochemistry, University of Catania, Catania, 95123 Italy; 4 Department of Human Sciences and Quality of Life Promotion, San Raffaele Roma Open University, 00166 Rome, Italy; 5 Department of Biomedical and Biotechnological Sciences, Laboratory of Translational Oncology and Functional Genomics, Section of General and Clinical Pathology and Oncology, University of Catania, Catania, 95123 Italy

**Keywords:** *Streptococcus agalactiae*, GBS neonatal infections, Serotypes, Antimicrobial susceptibility, Intrapartum antibiotic prophylaxis

## Abstract

*Streptococcus agalactiae* (also known Group B *Streptococcus* or GBS) represents the main pathogen responsible for early- and late-onset infections in newborns. The present study aimed to determine the antimicrobial susceptibility pattern and the capsular serotypes of GBS isolated in Eastern Sicily over 5 years, from January 2015 to December 2019. A total of 3494 GBS were isolated from vaginal swabs of pregnant women (37–39 weeks), as recommended by the Centers for Disease Control and Prevention. Capsular polysaccharide’s typing of GBS was determined by a commercial latex agglutination test containing reagents to serotypes I–IX. The antimicrobial resistance pattern of GBS was determined through the disk diffusion method (Kirby-Bauer) and the double-disk diffusion test on Mueller-Hinton agar plates supplemented with 5% defibrinated sheep blood, according to the guidelines of the Clinical and Laboratory Standards Institute. Serotypes III (1218, 34.9%) and V (1069, 30.6%) were the prevalent colonizers, followed by not typable (570, 16.3%) and serotypes Ia (548, 15.7%), Ib (47, 1.3%), II (40, 1.1%), and IV (2, 0.1%). All 3494 clinical isolates were susceptible to cefditoren and vancomycin. Resistance to penicillin, ampicillin, levofloxacin, clindamycin, and erythromycin was observed in 6 (0.2%), 5 (0.1%), 161 (4.6%), 1090 (31.2%), and 1402 (40.1%) of the strains, respectively. Most of erythromycin-resistant GBS (1090/1402) showed the cMLS_B_ phenotype, 276 the M phenotype, and 36 the iMLS_B_ phenotype. Our findings revealed a higher prevalence of serotype III and a relevant resistance rate, among GBS strains, to the most frequently used antibiotics in antenatal screening.

**Electronic supplementary material:**

The online version of this article (10.1007/s10096-020-03992-8) contains supplementary material, which is available to authorized users.

## Introduction

*Streptococcus agalactiae* is recognized by Lancefield classification as group B *Streptococcus* (GBS). This group includes nine historically known serotypes (Ia, Ib, II, III, IV, V, VI, VII, VIII) and a further (IX) of more recent identification [1, 2]. The discrimination of the different serotypes depends on type-specific capsular polysaccharides that constitute also a virulence factor, through which GBS eludes the host immune response [3, 4]. However, the expression of specific polysaccharides at the extracellular level is not the only invasion mechanism used by *S. agalactiae*. Indeed, analogously to other microorganisms [5], *S. agalactiae* is able to produce biofilm, a polysaccharide matrix that allows bacteria to hide from the immune system, favoring its persistence when environmental conditions are adverse [6].

Despite these pathogenic features, *S. agalactiae* is generally harmless for human health, being a commensal commonly present in the lower genital and gastroenteric tract of healthy women [7]. The screening for the identification of GBS becomes essential in pregnant women, due to a serious risk for newborn to contract the infection during birth. Data regarding the incidence of *S. agalactiae* infections in newborns are decidedly not encouraging. Accordingly, *S. agalactiae* is responsible for between 114,000 and 204,000 invasive cases and 147,000 stillbirths and infant deaths every year worldwide. Furthermore, the neonatal mortality rates, ranging from 10 to 15% and 40 to 58% in developed and developing countries, respectively, clearly confirm that vertical transmission of *S. agalactiae* still represents an emergency worldwide [8–10]. These numbers reflect a diffused and homogeneous vaginal and rectal GBS colonization among pregnant women worldwide, with some variations between the different regions of the world. In Europe, the variation of the colonization rate is around 7.1 to 16% [11, 12]. Interestingly, although *S. agalactiae* colonization rates are similar worldwide, serotype prevalence is geographically high variable [7]. In Europe and the USA, serotypes Ia, Ib, II, III, and V are the most common colonizers [1, 13, 14]. Serotypes VI and VIII appear typical of Japan [15], even if serotype VI was frequently isolated in Malaysia [16], Egypt [17], and Central Taiwan [18]. The importance to identify GBS serotypes lies in their different abilities to cause disease. According to this potential, neonatal invasive disease and meningitis are prevalently due to virulent serotype III, whereas serotypes Ia, Ib, II, III, and V are recognized as the most common isolated strains associated with GBS disease [7, 19]. Epidemiological studies, aimed to establish the predominant serotype/s in a specific country, could constitute a first step for the development of prevention strategies such as vaccines, which is being intensively worked on for several years [20]. Therefore, the prevention of GBS perinatal disease currently relies on maternal GBS colonization screening and intrapartum antibiotic prophylaxis (IAP), a pharmacologic approach diffusely adopted in the USA and Europe [21, 22].

The first-line agent for both IAP and treatment of GBS infected adults is penicillin. The universal use of this antibiotic is supported by the high susceptibility of GBS to β-lactams, in addition to an ostensible absence of penicillin resistance among GBS strains [1]. Concerning the latter issue, in 2008, Kimura et al. reported the first strains of GBS with reduced penicillin susceptibility [23]. Further studies confirmed that this reduced susceptibility is due to acquired mutations in penicillin-binding protein (PBP) domains [24–28]. It is worth noting that alternative therapeutic options for treating GBS infections must be designed also for penicillin-allergic patients [29]. In these cases, macrolides and lincosamides are considered valid substitute drugs [30, 31], although resistance to both antibiotic classes is well documented worldwide [32–34].

In light of all these considerations, it appears that the efficacy of IAP is strictly dependent on both the timely detection of GBS-infected pregnant women and the antibiotic resistance profile of the isolated strains. Interestingly, although the choice of antibiotic to prevent neonatal transmission is based on the local epidemiology of antimicrobial resistance of GBS, in Italy, these data are limited to northern and central areas, whereas no recent information is available for the southern regions [33, 35]. Specifically, data concerning colonization incidence, serotype prevalence, and antimicrobial susceptibility profiles of GBS among pregnant women in Sicily are still largely unknown, being related to small groups of patients and short-term observations. Therefore, in the present study, we proposed to fill this gap, by evaluating the antimicrobial susceptibility profiling of 3494 strains of *S. agalactiae*, describing also the prevalent serotypes in the Sicilian territory.

## Materials and methods

### Bacterial strains

During the years, the Microbiology Section of the Department of Biomedical and Biotechnological Sciences collected bacterial strains isolated from clinical specimens as support to routinely diagnostic activity, and 3494 of GBS strains were selected for this study. A total of 3494 GBS were isolated by clinicians from outpatients that attended public/private consulting rooms or obstetrics/gynecology clinics for routine screening during pregnancy (37–39 weeks), in the Eastern of Sicily, within 5 years comprised between January 2015 and December 2019. The samples were collected introducing sterile swabs in the middle third of the vaginal region and delivered to the bacteriology laboratory within half an hour. The swabs were anonymous and no information about patients was reported to the laboratory.

### Isolation, identification, and serotyping of GBS

The samples were processed as recommended by the Centers of Disease Control and Prevention (CDC) [31]. The swabs were inoculated in 3 ml of Todd-Hewitt Broth (THB) (bioMérieux SA, Marcy-l’Etoile, France) supplemented with nalidixic acid (15 μg/mL) (Sigma Aldrich, Italy) and gentamicin (8 μg/mL) (Sigma Aldrich, Italy). After incubation for 18–24 h at 35–37 °C under aerobic atmosphere, 10 μl of each broth was sub-cultured on 5% defibrinated sheep blood agar plates (SBA) (Becton Dickinson, Franklin Lakes, NJ, USA) and ChromID Strepto B Agar (STRB) (bioMérieux SA, Marcy-l’Etoile, France). The plates were incubated for 24 h at 35 °C in 5% CO_2_ (SBA) or under an aerobic atmosphere (STRB). GBS appeared as β-hemolytic gray to whitish-gray colonies on SBA [36] and pale pink to red colonies on STRB [37]. Identification of GBS was confirmed by an antigen detection latex agglutination test (Slidex® Strepto Plus B, bioMérieux SA, Marcy-l’Etoile, France). The isolated colonies on STRB were sub-cultured on SBA before performing agglutination assay. Capsular polysaccharides typing of GBS was determined by a commercial latex agglutination test containing reagents to serotypes I–IX (ImmuLex™ *Streptococcus* Antisera, SSI Diagnostica, Hillerød, Denmark) [38] according to the manufacturer’s recommendations. The bacteria were suspended in 5 ml of THB and incubated overnight. A 10 μl of each culture was mixed with 10 μl of specific antisera (serotypes I to IX), and agglutination was read after 5–10 s [39]. No agglutination after 30 s was considered a false positive. The strain was defined as not typable (NT) if the test failed to identify it into any serotype.

### Antimicrobial resistance testing of GBS

The antimicrobial resistance of GBS was determined through the disk diffusion method (Kirby-Bauer), according to the guidelines of the Clinical and Laboratory Standards Institute (CLSI) [40]. The clinical isolates were tested for susceptibility to seven different antibiotics, including penicillin (10 units), ampicillin (10 μg), cefditoren (5 μg), vancomycin (30 μg), levofloxacin (5 μg), clindamycin (2 μg), and erythromycin (15 μg) (Thermo Fisher Scientific Oxoid Ltd., Basingstoke, UK). Briefly, GBS colonies were suspended in 5 ml of sterile physiological saline, and the turbidity adjusted to a 0.5 McFarland standard, corresponding to a concentration of approximately 1.5 × 10^8^ CFU/ml. A sterile cotton swab was dipped in the bacterial suspension and swabbed over the surface of the Mueller-Hinton agar plates supplemented with 5% defibrinated sheep blood (MHAB) (bioMérieux SA, Marcy-l’Etoile, France). The antibiotic disks were placed on the plates and incubated for 20–24 h at 35 °C in a 5% CO_2_ atmosphere. After incubation, the zone of inhibition around the disks was measured by a calibrated ruler and interpreted using a standard chart (Table S1).

### Detection of macrolide-lincosamide-streptogramin B phenotypes

Macrolide-lincosamide-streptogramin B (MLS_B_) phenotypes were determined by the double-disk diffusion test on MHAB [40, 41]. Clindamycin and erythromycin disks were placed on agar surface 12 mm apart (edge to edge) [42]. After 24 h of incubation at 37 °C, the blunting of clindamycin inhibition zone proximal to the erythromycin disk was considered an inducible resistance (iMLS_B_), while resistance to both erythromycin and clindamycin with no blunting of clindamycin inhibition zone was suggested as a constitutive resistance (cMLS_B_). The M phenotype (efflux-mediated resistance) was resistant to erythromycin but susceptible to clindamycin, with no blunting of the inhibition zone around the clindamycin disk [41].

### Statistical analysis

To verify whether the difference in the trends of the distribution of the observed serotypes across 5 years is simply due to chance or not, we applied a Chi-square statistical test (Tables 1 and 2). The zero hypothesis (also called the null hypothesis) simply states that the observed difference—of whatever entity it is—is due to chance. This hypothesis, which may be true or false, will be accepted or rejected based on the result of an appropriate statistical test. When comparing two percentages or two proportions, the appropriate test is the Chi-square test. Furthermore, Cramer’s statistic was applied to data in Table 3, to determine the possible association between antimicrobial susceptibility profile and serotypes. Cramér’s V test is a measure of association between two nominal variables, giving a coefficient range between 0 (no relationship) and 1 (perfect relationship). It measures the association between variables using the medium squared deviation between the observed frequencies and the expected frequencies, expressed as a proportion of expected frequencies. Statistical tests were performed through the open-source environment R 3.6.3 [43].

**Table 1:** Distribution and statistical analysis of serological subtypes of GBS strains during the 5-year study period

	Molecular subtypes							
	Total no.	Ia	Ib	II	III	IV	V	No. of NT
2015	550	95 (17.3%)	10 (1.8%)	6 (1.1%)	120 (21.8%)	1 (0.2%)	124 (22.5%)	194 (35.3%)
2016	648	115 (17.7%)	15 (2.3%)	6 (1.0%)	148 (22.8%)	0 (0.0%)	160 (24.7%)	204 (31.5%)
2017	700	120 (17.1%)	7 (1.0%)	10 (1.4%)	235 (33.6%)	0 (0.0%)	320 (45.8%)	8 (1.1%)
2018	750	115 (15.3%)	10 (1.3%)	10 (1.3%)	335 (44.7%)	1 (0.1%)	250 (33.3%)	29 (4.0%)
2019	846	103 (12.2%)	5 (0.6%)	8 (0.9%)	380 (44.9%)	0 (0.0%)	215 (25.4%)	135 (16.0%)
5 years	3494	548 (15.7%)	47 (1.3%)	40 (1.1%)	1218 (34.9%)	2 (0.1%)	1069 (30.6%)	570 (16.3%)
*p* Value	2.12E–11	0.42	0.19	0.73	1.46E–44	0.56	6.78E–23	
χ^2^	70.13	3.86	6.08	2.00	211.20	3.00	110.14	

*NT* not typable

**Table 2:** Distribution and statistical analysis of resistant GBS strains during the 5-year study period

	Total no.	Pen-R	Amp-R	Cef-R	Van-R	Lev-R	Cli-R	Ery-R	Total no. R*
2015	550	0 (0.0%)	0 (0.0%)	0 (0.0%)	0 (0.0%)	8 (1.4%)	120 (21.8%)	138 (25.1%)	266 (48.3%)
2016	648	0 (0.0%)	0 (0.0%)	0 (0.0%)	0 (0.0%)	20 (3.1%)	130 (20.1%)	143 (22.1%)	293 (45.3%)
2017	700	1 (0.1%)	1 (0.1%)	0 (0.0%)	0 (0.0%)	35 (5.0%)	270 (38.6%)	378 (54.0%)	685 (97.8%)
2018	750	2 (0.3%)**	1 (0.1%)	0 (0.0%)	0 (0.0%)	38 (5.1%)	260 (34.7%)	337 (44.9%)	638 (85.1%)
2019	846	3 (0.3%)***	3 (0.3%)	0 (0.0%)	0 (0.0%)	60 (7.1%)	310 (36.6%)	406 (48.0%)	782 (92.3%)
5 years	3494	6 (0.2%)	5 (0.1%)	0 (0.0%)	0 (0.0%)	161 (4.6%)	1090 (31.2%)	1402 (40.1%)	2664 (76.2%)
*p* Value	2.12E–14	2.26E–01	1.99E–01	–	–	9.00E–07	2.20E–16	2.20E–16	2.20E–16
χ^2^	70.14	5.67	6.00	–	–	48.10	138.90	241.30	422.33

*Pen-R* penicillin-resistant, *Amp-R* ampicillin-resistant, *Cef-R* cefditoren-resistant, *Van-R* vancomycin-resistant, *Lev-R* levofloxacin-resistant, *Cli-R* clindamycin-resistant, *Ery-R* erythromycin-resistant. *Many strains were multidrug-resistant. **One strain with reduced penicillin susceptibility (PRGBS). ***One strain resistant to both macrolides and fluoroquinolones (multidrug-resistant)

**Table 3:** Serotype distribution and antimicrobial resistance to erythromycin and clindamycin of group B streptococci recovered from colonized pregnant women

			Phenotype			Molecular subtypes						
Total no.	Susceptibility pattern		M	iMLS_B_	cMLS_B_	Ia	Ib	II	III	IV	V	No. of NT
2092	Cli-S	Ery-S	0 (0.0%)	0 (0.0%)	0 (0.0%)	480 (22.9%)	28 (1.3%)	20 (1.0%)	762 (36.4%)	1 (0.1%)	590 (28.2%)	211 (10.1%)
1090	Cli-R	Ery-R	0 (0.0%)	0 (0.0%)	1090 (100%)	40 (3.7%)	19 (1.7%)	20 (1.8%)	372 (34.1%)	0 (0.00%)	329 (30.3%)	310 (28.4%)
276	Cli-S	Ery-R	276 (100%)	0 (0.0%)	0 (0.0%)	20 (7.2%)	0 (0.0%)	0 (0.0%)	84 (30.4%)	0 (0.0%)	122 (44.3%)	50 (18.1%)
36	Cli-R	Ery-S	0 (0.0%)	36 (100%)	0 (0.0%)	8 (22.2%)	0 (0.0%)	0 (0.0%)	0 (0.0%)	0 (0.0%)	28 (77.8%)	0 (0.0%)

*Cli-S* clindamycin-susceptible, *Cli-R* clindamycin-resistant, *Ery-S* erythromycin-susceptible, *Ery-R* erythromycin-resistant, *M* macrolide resistance phenotype, *iMLS*_*B*_ inductive macrolide-lincosamide-streptogramin B resistance, *cMLS*_*B*_ constitutive macrolide-lincosamide-streptogramin B resistance, *NT* not typable

## Results

**Fig. 1:**
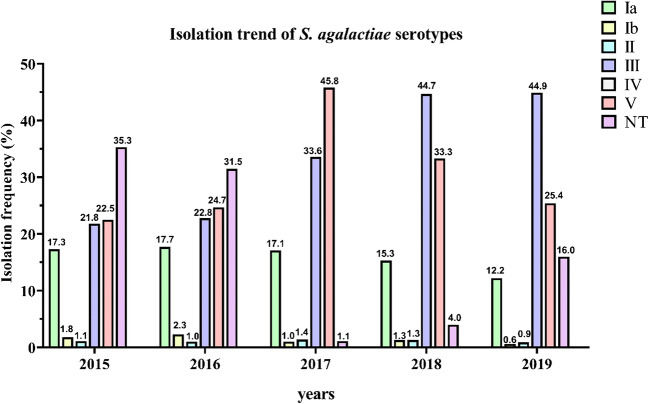
Isolation frequency of *S. agalactiae* (GBS) serotypes from 2015 to 2019. A total of 3494 GBS strains were isolated from vaginal swabs obtained by pregnant women at 37–39 weeks of gestation. Serotypes were identified by the agglutination test as described in the “Material and Methods” section. Histograms show the isolation frequency (percentage) of serotypes Ia, Ib, II, III, IV, and IV and not typable (NT) serotypes in the period between 2015 and 2019

**Fig. 2:**
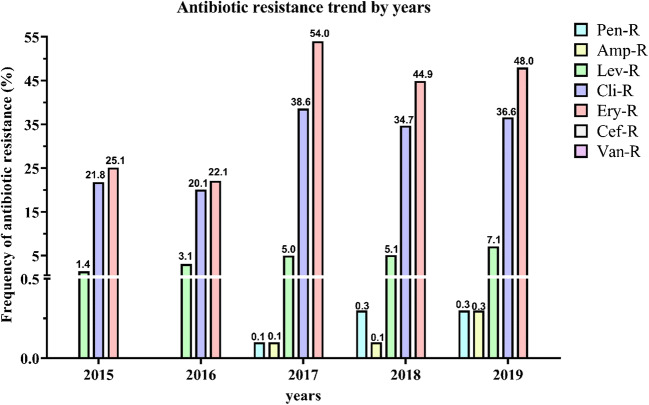
Antibiotic resistance pattern of *S. agalactiae* (GBS) strains from 2015 to 2019. Antibiotic resistance pattern of *S. agalactiae*, obtained by testing the antibiotic susceptibility of the isolated GBS strains to the most frequently used antibiotics in IAP. Histograms show the antibiotic resistance frequency (percentage) of GBS strains to penicillin, ampicillin, levofloxacin, clindamycin, erythromycin, cefditoren, and vancomycin in the period between 2015 and 2019

**Fig. 3:**
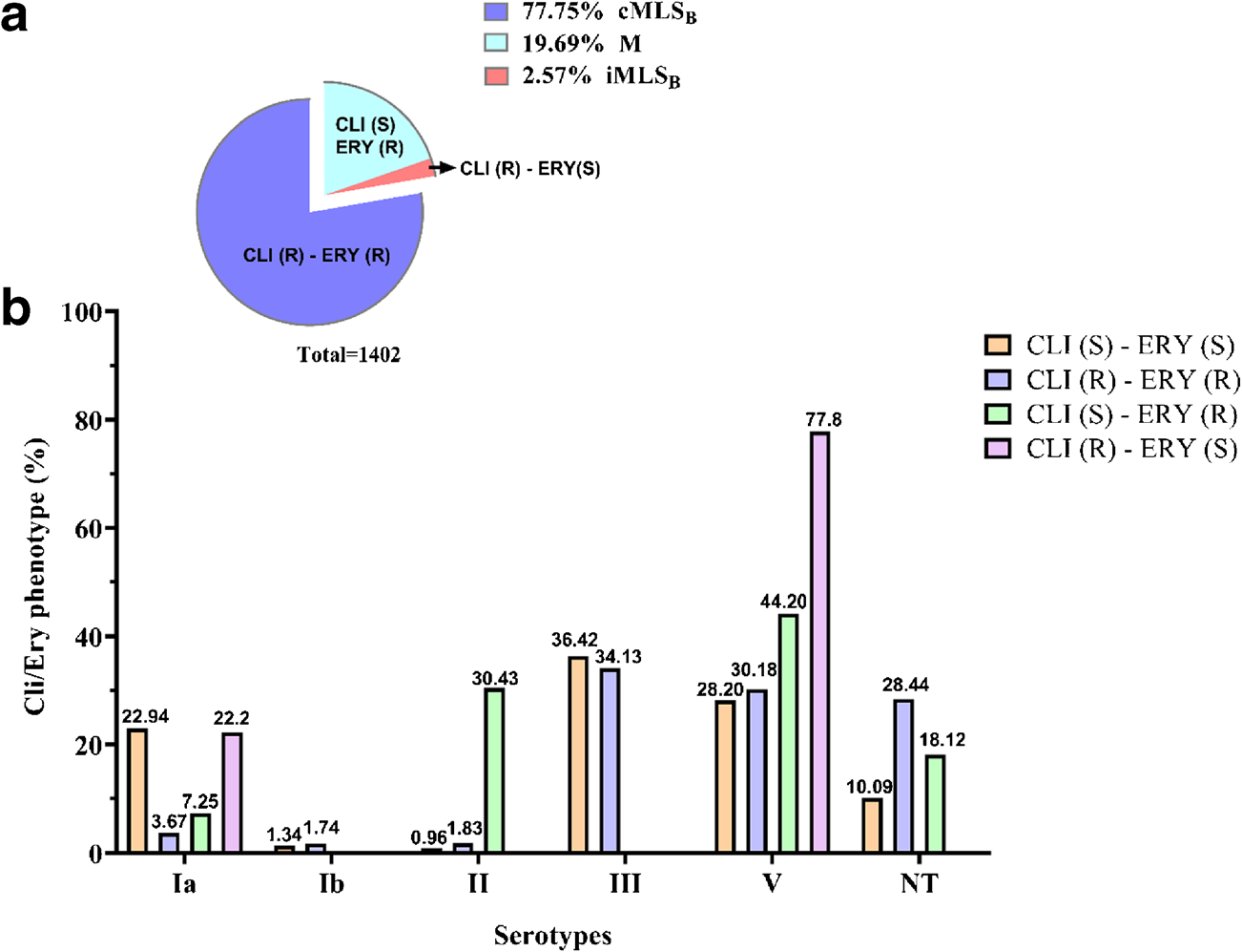
Correlation between GBS clindamycin/erythromycin phenotypes and serotypes. **a** Percentage of clindamycin-/erythromycin-resistant phenotypes among the1402 macrolide- and lincosamide- resistant GBS isolates. **b** Histograms show the frequency (expressed as a percentage) of each clindamycin/erythromycin phenotype among the identified GBS serotypes. Macrolide resistance phenotype (M); constitutive macrolide-lincosamide-streptogramin B resistance (cMLS_B_); inductive macrolide-lincosamide-streptogramin B resistance (iMLS_B_); clindamycin/erythromycin sensible (CLI (S) - ERY (S)); clindamycin-/erythromycin-resistant (CLI (R) - ERY (R)); clindamycin sensible and erythromycin-resistant (CLI (S) - ERY (R)); clindamycin-resistant and erythromycin sensible (CLI (R) - ERY (S)); *NT* not typable

The overall serotype frequencies of the 3494 GBS, isolated from vaginal swabs, were as follows: serotype Ia, 548 isolates (15.7%); serotype Ib, 47 (1.3%); serotype II, 40 (1.1%); serotype III, 1218 (34.9%); serotype IV, 2 (0.1%); serotype V, 1069 (30.6%); and not typable (NT), 570 (16.3%) (Table 1). Hence, serotype III was the predominant, followed by serotypes V, Ia, Ib, II, and IV. The trend of serotypes distribution throughout the period covered by the study is well illustrated in Fig. 1. No appreciable fluctuations in the frequencies of serotypes were observed, except for serotypes III and V, which increased (*p* value 1.46E–44) and decreased (*p* value 6.78E–23), respectively, from 2017 onwards. Accordingly, the high value of Chi-square test and the very low *p* value indicated that there was a statistically significant difference in the isolation percentages of the two serotypes (Table 1). Concerning the antimicrobial profile, the distribution of resistant GBS strains and the antibiotic resistance trend during the 5-year study are shown in Table 2 and Fig. 2, respectively. All the clinical isolates were susceptible to cefditoren and vancomycin, although very few strains resistant to penicillin and ampicillin appeared from 2017. Resistance to levofloxacin was observed in 161 (4.6%) strains, indicating no statistically significant fluctuation. A large proportion of GBS was resistant to erythromycin (1402, 40.1%) and clindamycin (1090, 31.2%), with a statistically significant increment from 2016. Regarding the penicillin-resistant isolates, the antibiotic resistance profile of these strains was reported in Table S2. In Table 3, it reported the correlation between serotype distribution and antimicrobial resistance to erythromycin and clindamycin. Most of the erythromycin-resistant GBS (1090/1402) showed the cMLS_B_ phenotype, 276 the M phenotype, and 36 the iMLS_B_ phenotype. Furthermore, the relationship between serotypes and phenotypes of macrolide- and lincosamide-resistant GBS strains was schematized in Fig. 3a–b. According to this analysis, the highest percentage of clindamycin-resistant and erythromycin sensible phenotype was found in serotype V (77.8%) followed by Ia (22.2%). The clindamycin sensible and erythromycin-resistant phenotype accounted for 44.20% of serotype V, 30.43% of II, 18.12% of NTs, and 7.25% of Ia. Similar percentages of clindamycin- and erythromycin-resistant phenotype were observed in serotypes III (34.13%) and V (30.18%), whereas the NTs represented the 28.44%, serotype Ia the 3.67%, serotype II the 1.83%, and serotype Ib the 1.74%. Finally, among clindamycin and erythromycin sensible phenotype, serotype III turned out to be the most frequent (36.42%), followed by serotype V (28.20%) and Ia (22.94%) and NTs (10.09%), (Fig. 3a–b). Statistically, Cramer’s V effect is considered small (*V* > 0.3), medium (*V* = 0.3), or large (*V* ≥ 0.50). In our case, the *V* value was of 0.247 which indicates a low association level among variables. Furthermore, the possible association between antimicrobial susceptibility profile and macrolide resistance phenotypes was also determined, obtaining a strong association level among variables (*V* value of 0.707).

## Discussion

*Streptococcus agalactiae* is an opportunistic agent, causing invasive infections mostly in immunocompromised patients [44]. This is particularly evident in newborns, in which a still precarious immune system favors the onset of severe GBS-related infections such as neonatal sepsis and meningitis [45, 46]. To date, ten GBS serotypes were identified [7] based on the antigenic specificity of capsular polysaccharide. The various serotypes are heterogeneously distributed among continents and are also characterized by a different grade of virulence, in turn, related to the severity of disease [47]. In the present study, serotype III was the most frequently recovered among the 3494 GBS-analyzed strains, accounting for 1218 (34.9%) of the isolated in 5 years, followed by serotypes V (1069, 30.6%) and Ia (548, 15.7%). It is well established that serotype III is the most virulent among GBS strains, causing the majority of late-onset infections [48]. Moreover, serotypes Ia, II, III, and V represent the main etiological agents of early onset-GBS infections and are largely diffused worldwide [49]. Therefore, the importance of surveillance programs, aimed to know the isolation frequency and distribution of the different GBS serotypes in a certain geographic area, is due to the tight correlation between serotypes and pathogenicity. Accordingly, data regarding the territorial prevalence of specific GBS serotypes could give an estimate of the risk for early- or late-onset neonatal diseases and could contribute to better define the preventive interventions (e.g., vaccine) at the local level. However, a proportion of GBS-isolated strains were not typing (NT) substantially due to the technical limitations of the serotyping method. The latex agglutination test is adopted by several European laboratories since it is rapid, easy to perform, and reproducible. Nevertheless, this serologic method often fails in typing the isolated strains due either to a low expression of the specific capsular polysaccharide or to the presence of variant forms of capsular structure that are not able to react with antibodies [38, 50]. Moreover, the performance of the agglutination test depends also on the quality of the antibodies used as well as to the laboratory expertise [51]. In a study conducted in Northern Italy, the serotyping of GBS strains by latex agglutination test highlighted 4 NTs out of a total of 58 macrolide-resistant GBS strains [33]. Given the detection failure of the serological test, molecular typing represents a valid alternative method for the identification of GBS serotypes. However, an Italian surveillance study showed that three GBS strains (1 out of 49 isolated from late-onset disease and 2 out of 320 from pregnant women) were not typable by either serologic or molecular typing methods [52]. Furthermore, Gherardi et al. provided evidence on the serotyping limit of molecular techniques, since 15 out of 91 GBS isolated from Italian hospitals still yielded the result NT by conventional phenotypic methods, failing to give any amplification of the *cps* locus by the molecular method [53]. On the other hand, the absence of some serotypes could not necessarily reflect an immunological method failure, rather than to arise by the differences in their geographical distribution. In fact, serotypes VI, VII, and VIII are the most common colonizers of Asia [54–56] and Egypt [17]; instead, serotype IX was isolated in Denmark and Australia [2, 57].

In the last few decades, the adoption of preventive strategies, including prenatal GBS colonization screening combined with intrapartum antibiotic prophylaxis, allowed to significantly decrease the incidence of GBS neonatal infections worldwide [21, 31]. The efficacy of antibiotic prophylaxis strictly depends on the choice of antimicrobial drugs. This aspect assumes fundamental importance for both the different antimicrobial susceptibility profile of GBS and the increasing development of resistance mechanisms against the most common antimicrobial agents used in pregnancy as prevention therapy [23–27]. Nevertheless, the current guidelines for intrapartum prophylaxis do not include antimicrobial susceptibility testing, which instead should be considered a routine test during the antenatal screening, able to ensure the efficacy of prophylactic therapy [31, 40].

In Italy, the evaluation of bacterial resistance against the main antibiotics used in IAP is limited to a few studies, most of which regard central [35, 58] and northern regions [59], whereas no data concerning the Sicily have been reported. Therefore, in our work, we evaluated the antimicrobial susceptibility profiling of 3494 clinical isolates of *S. agalactiae* collected during 5 years (from 2015 to 2019), in Sicily. To this purpose, we analyzed the antibiotic resistance of the GBS strains against the most frequently used antibiotics in preventing neonatal infections. It is well known that the antibiotic of choice in IAP is penicillin, for which very few cases of antibiotic resistance have been reported [23, 24]. Indeed, our results were substantially in agreement with this incidence, showing a low penicillin and ampicillin resistance rate among the tested strains, which became evident from 2017, but continuously maintained under 0.5% for the entire period considered. However, although low, the rates of penicillin and ampicillin resistance indicate a possible presence, in our territory, of GBS strains with reduced susceptibility to these β-lactams. Specifically, in our case, we found that among the six penicillin-resistant strains, one belonged to serotype Ia, two to III, and three to V. Interestingly, either of serotype III (strain number 124/846) showed resistance to three different antibiotic classes. Therefore, it is possible to define it as a multidrug-resistant (MDR) strain. Aminoacidic substitutions (V405A and/or Q557E) in penicillin-binding protein 2X (PBP2X) and other mutations on PBP2B and PBP1A domains, which confer β-lactam resistance, could be the basis of this reduced susceptibility [23–28]. However, since penicillin-resistant GBS strains were found in the different regions of the world, including the USA [24], Africa [34], Colombia [60], Japan [61], Central Italy [35], Scotland [62], and Canada [63], the question if these resistance phenotypes are due to sporadic mutations, independently acquired by some GBS strains, rather than to diffusion of β-lactam-resistant GBS clones, or even to both events, remains open.

Concerning the fluoroquinolone classes, we analyzed the efficacy of the antibiotic levofloxacin. Although the number of resistant strains constantly increased over the 5 years, going from only 8 (1.4%) cases in 2015 to 60 (7.1%) in 2019, this increment did not produce statistically significant fluctuation. Studies conducted in Northern (from January 2013 to June 2014) and Central (from 2010 to 2016) Italy revealed a levofloxacin resistance rate of 1.4% and 2.99%, respectively. Accordingly, the incidence of levofloxacin resistance of GBS is fairly constant in Italy. Furthermore, molecular investigations highlighted mutations in DNA gyrase and topoisomerase IV involved in fluoroquinolone resistance and consequently defined quinolone resistance-determining regions [58, 59]. Interestingly, all tested GBS strains were susceptible to cefditoren and vancomycin. The latter is an antimicrobial agent effective against a large variety of Gram-positive strains, including *S. agalactiae*, since it is able to inhibit the second stage of cell wall synthesis of these bacteria, leading to cell integrity loss [64].

Noteworthy are data regarding the number of erythromycin- and clindamycin-resistant isolates, which indicate high rates (1090 and 31.2% and 1402 and 40.1% in 5 years, respectively) of resistance to the most largely used antibiotics in cases of penicillin allergy, with a statistically significant increment from 2017. These results confirm the increasing incidence of macrolide and lincosamide resistance among GBS strains worldwide [1], suggesting that the use of these antibiotics should be opportunely preceded by routine susceptibility testing. Macrolide resistance derived from two different mechanisms, methylation of an adenine residue in 23S rRNA, a post traslational modification catalyzed by methylases encoded by *erm* genes, and active efflux. In the first case, the bacteria acquire MLS phenotype consisting of resistance to macrolide-lincosamide-streptogramin B antibiotics, which, in turn, can be constitutive (cMLS_B_) or inducible (iMLS_B_) [65]. The double-disk diffusion method allowed us to analyze MLS phenotypes among the GBS. The analysis of a possible correlation between GBS serotypes and phenotypes showed that serotype III was the most prevalent among both clindamycin and erythromycin susceptible and clindamycin- and erythromycin-resistant phenotypes, followed by serotype V. Moreover, all clindamycin- and erythromycin-resistant strains showed cMLS_B_ phenotypes, whereas the erythromycin-resistant GBS-isolated strains revealed M phenotypes, with a predominance of the serotype V(44.20%). It is well established the relationship between serotype V and erythromycin-resistant phenotype [66, 67]*.* Our findings are overall in agreement with these data and with a further study from Italy which showed that most of erythromycin-resistant GBS strains belonged to serotype V and almost all contained *erm*(B) gene [53]. Furthermore, it is worth to note that most of NT serotypes (28.44%) were clindamycin- and erythromycin-resistant phenotypes, indicating a prevalence of lincosamide- and macrolide-resistant GBS.

## Conclusion

Our study represents the first retrospective study conducted between January 2015 and December 2019 in Sicily, in which the serotype prevalence and the antibiotic susceptibility pattern of GBS strains, isolated from pregnant women, were described. The obtained results clearly showed that a large portion of GBS strains was resistant to the most common antimicrobial. Therefore, given that the eventuality to find penicillin-/ampicillin-resistant strains becomes increasingly concrete and that, in general, we are assisting to an increment of resistance rates to a wide variety of antimicrobial drugs, including the clindamycin and erythromycin, the main alternative antibiotics to β-lactams, our findings corroborate the necessity to verify possible antibiotic resistances of GBS strains, isolated during the antenatal screening, through antimicrobial susceptibility testing. The combination between these analyses and the introduction of surveillance programs that allow detecting the prevalence of specific serotypes in a certain territory could significantly contain the incidence of GBS-related neonatal infections.

## Electronic supplementary material

ESM 1 (DOCX 13 kb).

ESM 2 (DOCX 16 kb).
