# Supplementary material for: Streptococcus agalactiae in pregnant women: serotype and antimicrobial susceptibility patterns over five years in Eastern Sicily (Italy)
Source: Eur J Clin Microbiol Infect Dis. 2020 Jul 22;39(12):2387–96. doi: 10.1007/s10096-020-03992-8 (PMC7669783; doi:10.1007/s10096-020-03992-8)
Supplement: Supplementary file 1 — (DOCX 13 kb). [file 10096_2020_3992_MOESM1_ESM.docx]

**Table S1** CLSI breakpoints for Group B streptococci

|  | Zone diameter breakpoints (mm)^*^ | | |
| --- | --- | --- | --- |
| Antibiotic | Susceptible | Intermediate | Resistant |
| Pen | ≥24 | - | - |
| Amp | ≥24 | - | - |
| Cef^**^ | nr | nr | nr |
| Van | ≥17 | - | - |
| Lev | ≥17 | 14-16 | ≤13 |
| Cli | ≥19 | 16-18 | ≤15 |
| Ery | ≥21 | 16-20 | ≤15 |
| Pen, penicillin; Amp, ampicillin; Cef, Cefditoren; Van, vancomycin; Lev, levofloxacin; Cli, clindamycin; Ery, erythromycin; nr, not reported. ^*^Interpretive criteria CLSI M100-S25. ^**^ No CLSI breakpoints for cefditoren were reported. | | | |
