# Supplementary material for: Streptococcus agalactiae in pregnant women: serotype and antimicrobial susceptibility patterns over five years in Eastern Sicily (Italy)
Source: Eur J Clin Microbiol Infect Dis. 2020 Jul 22;39(12):2387–96. doi: 10.1007/s10096-020-03992-8 (PMC7669783; doi:10.1007/s10096-020-03992-8)
Supplement: Supplementary file 2 — (DOCX 16 kb). [file 10096_2020_3992_MOESM2_ESM.docx]

**Table S2** CLSI zone diameter breakpoints (mm) for penicillin-resistant GBS strains.

|  |  |  |  | Zone diameter breakpoints (mm)^*^ | | | | | | |
| --- | --- | --- | --- | --- | --- | --- | --- | --- | --- | --- |
| Year | Strain no.^**^ | Phenotype | Molecular subtype | Pen | Amp | Cef^***^ | Van | Lev | Cli | Ery |
| 2017 | 150/700 | cMLS_B_ | V | 22 (R) | 20 (R) | 26 (S) | 22 (S) | 20 (S) | 12 (R) | 10 (R) |
| 2018 | 320/750 | cMLS_B_ | V | 21 (R) | 24 (S) | 28 (S) | 20 (S) | 21 (S) | 13 (R) | 12 (R) |
|  | 470/750 | cMLS_B_ | III | 20 (R) | 20 (R) | 26 (S) | 19 (S) | 20 (S) | 10 (R) | 12 (R) |
| 2019 | 124/846 | cMLS_B_ | III | 16 (R) | 18 (R) | 26 (S) | 18 (S) | 11 (R) | 9 (R) | 8 (R) |
|  | 256/846 | cMLS_B_ | V | 20 (R) | 19 (R) | 27 (S) | 20 (S) | 19 (S) | 12 (R) | 12 (R) |
|  | 520/846 | cMLS_B_ | Ia | 21 (R) | 20 (R) | 28 (S) | 21 (S) | 20 (S) | 14 (R) | 10 (R) |
| Pen, penicillin; Amp, ampicillin; Cef, Cefditoren; Van, vancomycin; Lev, levofloxacin; Cli, clindamycin; Ery, erythromycin. ^*^Interpretive criteria CLSI M100-S25: S, susceptible; R, resistant. ^*^Strain numbers refer to an internal directory for clinical isolates. ^***^No CLSI breakpoints for cefditoren were reported. Group B streptococci with zone diameter breakpoint ≥24 mm were considered susceptible. | | | | | | | | | | |
